# Meta-analysis of radiofrequency ablation versus hepatic resection for small hepatocellular carcinoma

**DOI:** 10.1186/1471-230X-10-78

**Published:** 2010-07-09

**Authors:** Yanming Zhou, Yanfang Zhao, Bin Li, Donghui Xu, Zhengfeng Yin, Feng Xie, Jiamei Yang

**Affiliations:** 1 Department of Hepato-Biliary-Pancreato-Vascular Surgery, the First affiliated Hospital of Xiamen University, Xiamen, China; 2 Department of Health Statistics, Second Military Medical University, Shanghai, China; 3 Department of Molecular Oncology, Eastern Hepatobiliary Surgery Hospital, Second Military Medical University, Shanghai, China; 4 Department of Special Treatment, Eastern Hepatobiliary Surgery Hospital, Second Military Medical University, Shanghai, China

## Abstract

**Background:**

There is no clear consensus on the better therapy [radiofrequency ablation (RFA) versus hepatic resection (HR)] for small hepatocellular carcinoma (HCC) eligible for surgical treatments. This study is a meta-analysis of the available evidence.

**Methods:**

Systematic review and meta-analysis of trials comparing RFA with HR for small HCC published from 1997 to 2009 in PubMed and Medline. Pooled odds ratios (OR) with 95% confidence intervals (95% CI) were calculated using either the fixed effects model or random effects model.

**Results:**

One randomized controlled trial, and 9 nonrandomized controlled trials studies were included in this analysis. These studies included a total of 1411 patients: 744 treated with RFA and 667 treated with HR. The overall survival was significantly higher in patients treated with HR than in those treated with RFA at 3 years (OR: 0.56, 95% CI: 0.44-0.71), and at 5 year (OR: 0.60, 95% CI: 0.36-1.01). RFA has a higher rates of local intrahepatic recurrence compared to HR (OR: 4.50, 95% CI: 2.45-8.27). In the HR group the 1, 3, and 5 years disease -free survival rates were significantly better than in the HR-treated patients (respectively: OR: 0.54, 95% CI: 0.35-0.84; OR: 0.44, 95% CI: 0.28-0.68; OR: 0.64, 95% CI: 0.42-0.99). The postoperative morbidity was higher with HR (OR: 0.29, 95% CI: 0.13-0.65), but no significant differences were found concerning mortality. For tumors ≤ 3 cm HR did not differ significantly from RFA for survival, as reported in three NRCTs .

**Conclusions:**

HR was superior to RFA in the treatment of patients with small HCC eligible for surgical treatments, particularly for tumors > 3 cm. However, the findings have to be carefully interpreted due to the lower level of evidence.

## Background

Hepatocellular carcinoma (HCC) is the fifth most common cancers in the world, with an estimated 500,000 deaths per year [1]. Advances in diagnostic imaging and widespread application of screening programs in high-risk populations have allowed detection of small HCC, which can be curable by partial hepatic resection (HR), liver transplantation, or local ablation therapies. Out of these, liver transplantation, which offers the potential to both resect the entire potentially tumor-bearing liver and eliminate the cirrhosis, achieves the best results but can be offered only to a minority of patients because of the shortage of donors and high cost [2]. Therefore, HR has generally been accepted as the first treatment of choice for HCC in many centers. Nevertheless, the associated cirrhosis limits the extent of surgery and thus increases the risk of postoperative liver failure. So, many nonsurgical ablative methods have been developed, such as cryoablation [3], percutaneous ethanol injection (PEI) [4], acetic acid injection [5], radiofrequency ablation (RFA) [6], microwave coagulation [7], and transcatheter arterial chemoembolization (TACE) [8].

Among these therapies, RFA is a promising and recently developed ablation technique. It induces deep thermal injury in hepatic tissue while sparing the normal parenchyma. Its basic principle includes generation of high-frequency alternating current which causes ionic agitation and conversion to heat, with subsequent evaporation of intracellular water which leads to irreversible cellular changes, including intracellular protein denaturation, melting of membrane lipid bilayers, and coagulative necrosis of individual tumor cells. RFA was recommended as the best treatment option for patients with early stage HCC who are not suitable for resection or transplantation in addition to PEI in the 2005 practice guidelines issued by the American Association for the Study of Liver Diseases [9]. Recently, a meta-analysis of randomized controlled trial (RCT) showed that RFA ablation is superior to PEI in the treatment of patients with relatively preserved liver function and early-stage non-surgical HCC with respect to survival and local control of the disease [10]. In contrast, whether RFA or HR is the better treatment for HCC eligible for surgical treatments has long been debated. Several RCT and non-randomized controlled trial (NRCT) have been published in an attempt to answer the above question. What follows is a meta-analysis of these studies.

## Methods

### Study selection

Electronic searches were performed of the PubMed and Medline from January 1997 to November 2009. The following Mesh search headings were used: "radiofrequency ablation," "hepatocellular carcinoma". Reference lists of all retrieved articles were manual searched for additional studies. No language restrictions were made.

### Data extraction

Two reviewers (BL and DX, respectively) independently extracted the following parameters from each study: 1) first author and year of publication; 2) number of patients, patients characteristics, study design; and lastly, 3) treatment outcome. All relevant text, tables and figures were reviewed for data extraction. Discrepancies between the two reviewers were resolved by discussion and consensus.

### Criteria for inclusion and exclusion

For inclusion in the meta-analysis, a study had to fulfil the following criteria: 1) compare the initial therapy effects of RFA and HR for the treatment of small HCC, regardless of the etiology of liver disease, cirrhotics status, or differences in viral hepatitis; 2) report on at least one of the outcome measures mentioned below; 3) clearly document indications for RFA and HR; 4) In dual (or multiple) studies were reported by the same institution and/or authors, either the one of higher quality or the most recent publication was included in the analysis. The primary endpoints were overall survival at 1, 3, and 5 years, and local recurrence. The secondary endpoints were disease-free survival at 1, 3, and 5 years, morbidity, and mortality.

Small HCC are defined according to the criteria developed by Yao et al. [11] from the University of California, San Francisco, a single HCC nodule of up to 6.5 cm, or with up to 3 lesions, the largest of which is no larger than 4.5 cm.

Abstracts, letters, editorials and expert opinions, reviews without original data, case reports and studies lacking control groups were excluded. The following studies were also excluded: 1) those dealing with unresectable HCC or HCC recurrence after hepatectomy; 2) those with no clearly reported outcomes of interest; 3) those evaluating patients with cholangiocellular carcinomas or liver metastases.

### Statistical methods

The meta-analysis was performed using the Review Manager (RevMan) software, version 4.2.7. All analyses were performed on dichotomous outcomes. Pooled odds ratios (OR) with 95% confidence intervals (95% CI) were calculated using either the fixed effects model or random effects model. Heterogeneity was evaluated by χ2 and I^2^. I^2 ^< 25% and I^2 ^> 50% reflect small and large inconsistency, respectively. *P *< 0.05 was considered significant.

## Results

### Selection of trials

After initial screening, 19 potentially relevant clinical trials of HCC were identified. Of these, three trials did not separately analyze the results of RFA from the other local therapies [12-14], two trials including patients with unresectable HCC [15,16], one trial comparing resection with RFA combined with preceding TACE [17], all 6 studies were excluded. Three trials were also excluded from the analysis, as no information concerning 3 or 5-year overall survival was provided in these trials [18-20]. Finally, a total of 10 studies (1 RCT and 9 NRCTs) [21-30] published between 2004 and 2009 matched the selection criteria and were therefore included. Eight of these studies used percutaneous RFA [21-28], and the remaining 2 studies used laparoscopic RFA [29,30]. These studies included a total of 1411 patients: 744 treated with RAF and 667 treated with HR. The mean age ranged from 49.2 ± 9.9 to 69.4 ± 9.1 years. Male: female ratio in the pooled data was 2.4: 1. 84.7% of patients were in Child-Pugh class A. Most patients (91.0%, 1139/1251) had a single tumor. The median/mean tumor size (cm) ranged from 1.98 to 3.65. Median/mean duration of follow-up ranged from 21.9 to 43 months (Table 1).

**Table 1:** Characteristics of included trials

Author/(year)	Design	Inclusion criteria	Treatment	No. of patients	M/F	Mean age (years)	Mean tumor size(cm)	Mean AFP (ng/ml)	Child-Pugh A/B	Mean follow-up (months)
Vivarelli	NRCT	(1)cirrhosis; (2)Child-Pugh class A or B	RFA	79	67/12	67.8 ± 8.7	--	> 20 (n = 26)	43/36	15.6 ± 11.7
(2004)			HR	79	57/22	65.2 ± 8.2	--	> 20 (n = 43)	70/9	28.9 ± 17.9
Hong	NRCT	(1) patients with liver cirrhosis whose Child-Pugh score is 5 or those without cirrhosis; (2)solitary tumor < 4 cm; (3) no vascular invasion; (4) no previous treatment	RFA	55	41/14	59.1 ± 9.6	2.4 ± 0.6	447.3 ± 981.3	55/0	22.7*
(2005)			HR	93	69/24	49.2 ± 9.9	2.5 ± 0.8	644.0 ± 1745.6	93/0	25.5
Cho	NRCT	(1)Single or multiple (≤ 3 nodules) ≤ 5 cm; (2) Pugh-Child Class A; (3) no extrahepatic metastasis	RFA	99	76/23	58	3.1 ± 0.8	--	99/0	23 ± 9.4
(2005)			HR	61	48/13	57	3.4 ± 1	--	61/0	21.9 ± 9 .8
Montorsi	NRCT	(1) Cirrhosis (2)Solitary tumor ≤ 5 cm. single lesion; (3) Child A-B class; (4) segmental or subsegmental resection possible; (5) no previous treatment	RFA	58	43/15	67 ± 6	--	377.7 ± 1051.8	40/18	25.7 ± 16.7
(2005)			HR	40	33/7	67 ± 9	--	361.3 ± 1026.2	32/8	22.4 ± 17.5
Chen	RCT	(1) age 18 to 75 years; (2) solitary tumor ≤ 5 cm; (3)no extrahepatic metastasis; (4) Pugh-Child Class A, with no history of ncephalopathy, ascites refractory to diuretics, or variceal bleeding; (5) ICG-R15 < 30%; (6) no previous treatment	RFA	71	56/15	51.9 ± 11.2	--	> 200 (n = 31)	71/0	27.9 ± 10.6
(2006)			HR	90	75/15	49.4 ± 10.9	--	> 200 (n = 30)	90/0	29.2 ± 11.9
Lupo	NRCT	(1)Single tumor 3-5 cm; (2) no previous treatment; (3) no extrahepatic metastasis; (4) Child-Pugh class A or B	RFA	60	47/13	68	3.65*	> 200 (n = 3)	44/16	27 ± 18.7
(2007)			HR	42	33/9	67	4	> 200 (n = 5)	28/14	31.3 ± 24.3
Guglielmi	NRCT	(1)Cirrhosis; (2)Single or multiple (≤ 3 nodules) ≤ 6 cm; (3)Child-Pugh class A or B	RFA	109	88/21	--	--	> 200 (n = 43)	64/45	23
(2008)			HR	91	73/18	--	--	> 200 (n = 34)	69/22	32
Hiraoka	NRCT	(1)Single tumor ≤ 3 cm;	RFA	105	76/29	69.4 ± 9.1	1.98	114.5	79/26	847$
(2008)		(2) Child-Pugh class A or B	HR	59	44/15	62.4 ± 10.6	2.27	427.8	54/5	927
Abu-Hila	NRCT	(1)Cirrhosis;	RFA	34	27/7	65	3*	--	27/7	30*
(2008)		(2)Single tumor 1-5 cm.	HR	34	26/8	67	3.8	--	25/9	43
Santambrogio	NRCT	(1)Single tumor < 5 cm; (2)Child-Pugh class A	RFA	74	59/15	68 ± 7	2.66	9	74/0	38.2 ± 28.4
(2009)			HR	78	55/23	68 ± 8	2.91	8	78/0	36.2 ± 23.5

RCT: randomised controlled trial. NRCT: non-randomized controlled trial. AFP: alpha-fetoprotein. RFA: radiofrequency ablation. HR: hepatic resection. M: male. F: female. *: median. $: Day.

### Overall survival

**Figure 1:**
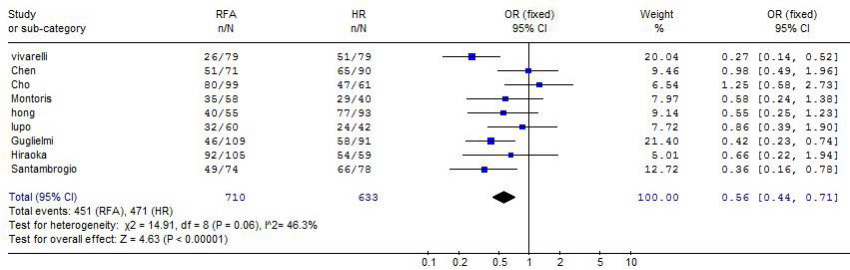
**Results of the meta-analysis on overall survival at 3 years**.

There was no significant difference in overall survival between the two groups at 1 year (all trials reported this data, OR: 0.84, 95% CI: 0.58-1.21), and at 2 years (four trials reported this data, OR: 0.80, 95% CI: 0.50-1.27), whereas the difference was significant and favourable to HR group at 3 years (nine trials reported this data, OR: 0.56, 95% CI: 0.44-0.71) ( Figure 1), and at 5 year (five trials reported this data, OR: 0.60, 95% CI: 0.36-1.01) (Table 2).

**Table 2:** Summary of the results on the efficacy and safety of RFA versus HR in the management of small HCC

Variables	No. of studies	Results		OR (95% CI)	*P*-value	**I**^**2**^
	furnishing data	RFA	HR			
**Efficacy:**						
Overall survival						
1 year	10^21-30^	89.7%	91.3%	0.84 (0.58, 1.21)	0.34	0%
2 years	4^23-25, 29^	79.3%	82.2%	0.80 (0.50, 1.27)	0.35	0%
3 years	9^21-28, 30^	63.5%	74.4%	0.56 (0.44, 0.71)	< 0.001	46.3%
5 years	5^26-30^	41.3%	51.9%	0.60 (0.36, 1.01)	0.05	61.6%
Intrahepatic recurrence						
Local recurrence	5^22-24, 29, 30^	19.0%	4.2%	4.50 (2.45, 8.27)	< 0.001	10.7%
Distant recurrence	5^22-24, 29, 30^	39.6%	38.8%	1.16 (0.83, 1.61)	0.38	27.1%
Disease-free survival						
1 year	9^21-29^	68.8%	80.3%	0.54 (0.35, 0.84)	0.006	59.2%
2 years	3^24, 25, 29^	49.6%	74.3%	0.34 (0.21, 0.55)	< 0.001	0%
3 years	8^21-28^	34.9%	54.4%	0.44 (0.28, 0.68)	< 0.001	66.7%
5 years	4^25-28^	18.4%	23.8%	0.64 (0.42, 0.99)	0.05	47.2%
**Safety:**						
Morbidity	6^23, 25, 26, 27, 29, 30^	9.6%	32.5%	0.29 (0.13, 0.65)	0.003	72.7%
Mortality	10^21-30^	0.1%	0.8%	0.36 (0.10, 1.27)	0.11	0%

RFA: radiofrequency ablation.HR: hepatic resection. OR: odds ratios.

### Intrahepatic recurrence

**Figure 2:**
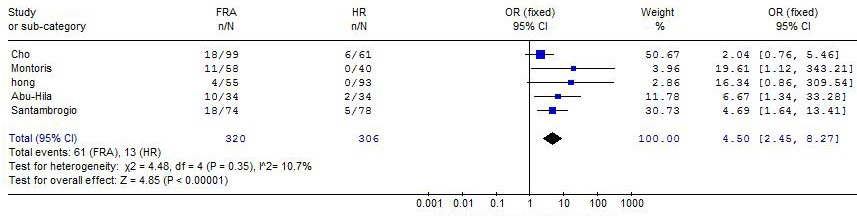
**Results of the meta-analysis on local recurrence rate**.

The pooled analysis of the 5 studies furnishing data showed that RFA has a higher rates of local intrahepatic recurrence compared to HR (OR: 4.50, 95% CI: 2.45-8.27) (Figure 2). No differences were found between the two groups with respect to the distant intrahepatic recurrence (Table 2).

### Disease-free survival

As Table 2 shows, our results indicate that 1, 3 and 5 year disease-free survival rates were significantly higher in the HR group.

### Safety

The rate of postoperative morbidity was significantly higher in patients treated with HR than in those treated with RFA (six trials reported this data, OR: 0.29, 95% CI: 0.13-0.65), but no differences were found between the two groups with respect to postoperative mortality (all trials reported this data, OR: 0.36, 95% CI: 0.10-1.27). There were 1 death reported in the RFA group, and 6 in the HR group, giving a mean mortality rate of 0.1 per cent and 0.8 per cent, respectively (Table 2).

### Comparison between the two groups in HCCs ≤ 3 cm

Three NRCTs compared RFA versus HR for patients with tumors ≤ 3 cm. Of these, two studies included only patients with single tumor [27,28]. There was no significant difference in overall and disease -free survival between the groups at 1, 3, and 5 years in each included study (Table 3). In the further analysis, survival of patients with single or multiple tumors was similar in the two treatment groups, as reported by Vivarelli et al. [21]. Thus, we did not perform a meta-analysis of these studies.

**Table 3:** Studies comparing RFA versus HR for HCC ≤ 3 cm

Author	Treatment	No. of patients	Survival (%)			Disease-free survival (%)			*P*-value
			1-year	3-year	5-year	1-year	3-year	5-year	
Vivarelli	RFA	22	89	--	50	70	34	--	NS
	HR	21	89	--	79	84	67	--	
Guglielmi	RFA	32	91	50	29	72	36	36	NS
	HR	31	89	78	54	80	58	19	
Hiraoka	RFA	105	95.1	87.8	59.3	87.5	58.7	24.6	NS
	HR	59	98.1	91.4	59.4	91.4	64.3	22.4	

RFA: radiofrequency ablation.HR: hepatic resection. NS: not significant

## Discussion

Meta-analysis, a quantitative technique for therapeutic evaluation, may be used when controversy persists after several trials. Although meta-analysis has traditionally been applied and is best confined to RCT, meta-analytical techniques using NRCT might be a valid method in some clinical settings in which either the number or the sample size of RCT is insufficient [31].

This meta-analysis shows that in patients with small HCC the treatment with HR is superior to treatment with RFA. HR had a significant better survival in terms of overall survival at 3, and 5 years, and disease-free survival at 1, 3, and 5 years. The difference at 5 years overall survival was marginally significant due to the number of studies furnishing combinable data on this outcome is limited. Although the only RCT shows nearly equivalent survival with the two treatments, however, in this study 19 of 90 patients (21%) who were randomized for RFA converted to HR [25].

High rate of intrhepatic recurrence after ablation therapies and/or surgical resection is the main cause of late death of patients with HCC. In current study, local recurrence was found to be more frequent after RFA than after HR. Local recurrences after RFA may be attributable to insufficient ablation of the primary tumor and/or the presence of tumor venous invasion in the adjacent liver. Surgical resection could remove the primary tumor and venous tumor thrombi [12]. This may explain the better outcomes following HR.

As regards the distant recurrence, no differences were found between the two groups. This fnding is reasonable because the occurrence of distant recurrence is correlated with the host factors and initial tumor factors [32], and the treatment does not affect this outcome.

Although the overall rate of postoperative morbidity was 32.5% in patients treated with HR, which is significantly higher than in those treated with RFA. However, these rates do not translate into an increase in mortality.

The results of this meta-analysis should be interpreted with caution for several reasons. First, the majority of data in the present study comes from NRCTs, and the overall level of clinical evidence is low. Second, there are important heterogeneity between two groups. This could be due to a bias in patient selection in several series in terms of patient demographics and tumor characteristics. Patients undergoing RFA had a few less favourable characteristics such as old age, multiple nodules, severe chronic liver disease, high serum level of alpha-fetoprotein and aspartate aminotransferase level [22,25,27,28]. However, Guglielmi et al. [27] found that these biases did not influence the statistical analysis in the subgroups and in the multivariate analyses. We applied a random effect model to take between study variation into consideration. This does not necessarily rule out the effect of heterogeneity between studies, but one may expect a very limited influence.

Third, RFA can be performed by percutaneous, laparoscopic or open approaches. Laparoscopic and open approaches increase the chance of detection of unknown intrahepatic and extrahepatic tumors because they allow complete abdominal exploration and intraoperative ultrasound assessment. The additional advantages of open and laparoscopic approaches are the accurate placement of electrodes and the possible treatment of tumors in percutaneously inaccessible areas of the liver and tumors in close proximity to or invading the adjacent organs [6]. On the other hand, several different RFA systems were used in the treatment centers, such as RITA Medical System (Mountain View, CA), Radionics (Burlington, MA) and Valleylab (Boulder, CO). Different approaches and RFA systems would also impact on the pooling of data and interpretation of results. Unfortunately, we failed to find any study that compared the outcomes of different approaches and RFA systems on therapy efficacy of HCC. Thus, we were not able to assess the influence of these factors.

Vascular invasion is a well-established prognostic indicator of HCC. Considering that vascular invasion was less frequent in tumors ≤ 3 cm [33], this may explain equivalent survival outcome between two groups, as reported in 3 trials. RFA appears to be an effective alternative treatments modality for HCC ≤ 3 cm. However, the only three studies containing 270 patients make the interpretation of these results insufficient. Recently, in a large, prospective study from the Liver Cancer Study Group of Japan, 7185 patients with no more than 3 tumors (≤ 3 cm) were classified into either a resection (n = 2857), RFA (n = 3022), or PEI group (n = 1306). The comparison of all three groups showed that surgical resection may be superior to ablation with respect to the prevention of recurrence [20]. Thus, the efficacy compared to HR or ablation in the treatment for HCC ≤ 3 cm still is a matter of discussion and further studies are required to provide more adequate data answer this question.

## Conclusions

HR was superior to RFA in the treatment of patients with small HCC eligible for surgical treatments, particularly for tumors > 3 cm. However, the findings have to be carefully interpreted due to the lower level of evidence. Further RCT are needed to define the exact value of HR and RFA for small HCC, even for tumors ≤ 3 cm.

## Competing interests

The authors declare that they have no competing interests.

## Authors' contributions

YZhou participated in the design and coordination of the study, carried out the critical appraisal of studies and wrote the manuscript. BL, DX, ZY and developed the literature search, carried out the extraction of data, assisted in the critical appraisal of included studies and assisted in writing up. YZhao and FX carried out the statistical analysis of studies. JY interpreted data, corrected and approve the manuscript. All authors read and approved the final manuscript.

## Pre-publication history

The pre-publication history for this paper can be accessed here:

http://www.biomedcentral.com/1471-230X/10/78/prepub
